# A novel *SALL1* C757T mutation in a Chinese family causes a rare disease --Townes-Brocks syndrome

**DOI:** 10.1186/s13052-024-01691-0

**Published:** 2024-06-24

**Authors:** Yunqian Chi, Yi Yao, Futao Sun, Wenhong Zhang, Zihan Zhang, Yunhe Wang, Wei Hao

**Affiliations:** 1 Department of Neonatology, Shandong Provincial Hospital Affiliated to Shandong First Medical University, Jinan, 250021 Shandong Province PR China; 2 Basic Medical College, Guangxi Medical University, Nanning, 530021 Guangxi Province PR China; 3 Department of Pediatric Surgery, Shandong Provincial Hospital Affiliated to Shandong First Medical University, Jinan, 250021 Shandong Province PR China; 4 Shandong Provincial Hospital, Shandong First Medical University, Jinan, 250021 Shandong Province China

**Keywords:** *SALL1*, Townes-Brocks syndrome, Whole-exome sequencing, Anorectal malformation, Triplet pregnancy

## Abstract

**Background:**

Townes-Brocks syndrome (TBS) is a rare genetic disorder characterized by imperforate anus, dysplastic ears, thumb malformations, and other abnormalities. Previous studies have revealed that mutations in the *SALL1* gene can disrupt normal development, resulting in the characteristic features of Townes-Brocks syndrome. Spalt-like transcription factors (SALLs) are highly conserved proteins that play important roles in various cellular processes, including embryonic development, cell differentiation, and cell survival. Over 400 different variants or mutations have been reported in the *SALL1* gene in individuals with TBS. Most of these variants lead to the formation of premature termination codons (PTCs), also known as nonsense mutations. The majority of these PTCs occur in a specific region of the *SALL1* gene called the “hotspot region”, which is particularly susceptible to mutation.

**Methods:**

In this study, we conducted whole-exome sequencing on a three-generation Chinese family with anorectal malformations.

**Results:**

We identified a novel heterozygous mutation (chr16:51175376:c.757 C > T p.Gln253*) in the *SALL1* gene. Molecular analysis revealed a heterozygous C to T transition at nucleotide position 757 in exon 2 of the *SALL1* (NM_002968) gene. This mutation is predicted to result in the substitution of the Gln253 codon with a premature stop codon (p.Gln253*). The glutamine-rich domain forms a long alpha helix, enabling the mutant protein to interact with the wild-type SALL1 protein. This interaction may result in steric hindrance effects on the wild-type SALL1 protein.

**Conclusions:**

Our findings have expanded the mutation database of the *SALL1* gene, which is significant for genetic counseling and clinical surveillance in the affected family. Furthermore, our study enhances the understanding of Townes-Brocks syndrome and has the potential to improve its diagnosis and treatment.

## Introduction

Townes-Brocks syndrome (TBS, OMIM: #107,480) is a congenital genetic disorder characterized by the triad including malformations of anus, external ears and thumbs. Other features include hearing loss, foot deformity, renal insufficiency with or without associated urogenital abnormalities, and congenital heart disease, including atrial septal defect, ventricular septal defect, tetralogy of Fallot, truncus arteriosus (type A or type B), pulmonary valve atresia, and persistent ductus arteriosus [1–4]. Dysplastic ears (overfolded superior helices, microtia), thumb abnormalities (preaxial polydactyly, triphalangeal thumbs, and hypoplastic thumbs without hypoplasia of the radius), lower extremities deformities (clubfoot, overlapping toes, syndactyly, and missing toes), vaginal aplasia with bifid uterus, bifid scrotum, cryptorchidism are also observed. Additionally, some individuals with TBS may experience developmental delay or intellectual disability. Currently, the diagnosis of TBS requires the presence of three major or two major and minor features [3]. The major criteria for diagnosing TBS include an imperforate anus (found in 84% of cases), abnormally shaped ears (found in 87% of cases), and thumb malformations (found in 89% of cases). Minor features associated with TBS may include renal abnormalities, hearing loss, heart defects, genitourinary and limb abnormalities, and anal stenosis [5]. Early identification of TBS is fundamental to support child health and development (e.g. hearing aids, early surgical intervention) and to provide genetic counseling [6]. TBS is an autosomal dominant genetic disease due to *SALL1* gene mutation [7]. 

Spalt-like transcription factors, also known as SALLs, belong to a family of zinc finger transcription factors that play important roles in various biological processes, including embryo development, apoptosis, angiogenesis, and metastasis [8]. SALL1 is a zinc finger protein believed to function as a transcription factor. It consists of three exons that encode a zinc finger protein responsible for transcriptional repression. The protein contains four highly conserved double zinc finger domains, with a single C2H2 motif attached to the second domain. SALL1 protein is exclusively found in pericentromeric heterochromatin. Most *SALL1* mutations results in alteration in the N-terminal third of the encoded protein, causing truncation mostly at C2H2 and N-terminal transcriptional repressor domains [9]. To date, over 400 different *SALL1* variants with overlapping phenotypes have been reported in TBS patients [3]. Indeed, the precise mechanism by which specific variants or mutations in the genes associated with TBS lead to the development of the disorder is not fully understood.

Here, we identified a novel heterozygous mutation (c.757 C > Tp.Gln253*) in *SALL1* through whole-exome sequencing of a family presenting a congenital anal agenesis. In addition to this, two family members presented with mild hearing impairment. In silico study was performed to examine the pathogenicity of the variant. The results showed that the glutamine-rich domain forms a long alpha helix, allowing the mutant protein to interact with the wild-type SALL1 protein between the two glutamine-rich domains of both wild-type and mutant SALL1 proteins that might exert steric hindrance effects, altering thus its function. This finding is consistent with previous reports. These results contribute to expanding the spectrum of *SALL1* mutations and provide a reference for clinical practice.

## Patients and methods

### Case presentation

The proband was a 1-day-old female newborn and her two sisters born from twin gestation with known anorectal malformations (ARMs) from a townlet in China. Their mother was 33 years old and previously had a spontaneous birth. During this spontaneous pregnancy, transabdominal ultrasound examination diagnosed triplet gestation. Nuchal Translucency (NT) and amniocentesis were not performed during pregnancy. No abnormalities were found in the Oral Glucose Tolerance Test、quantity of amniotic liquid、fetal heart and blood pressure monitoring.

**Fig. 1:**
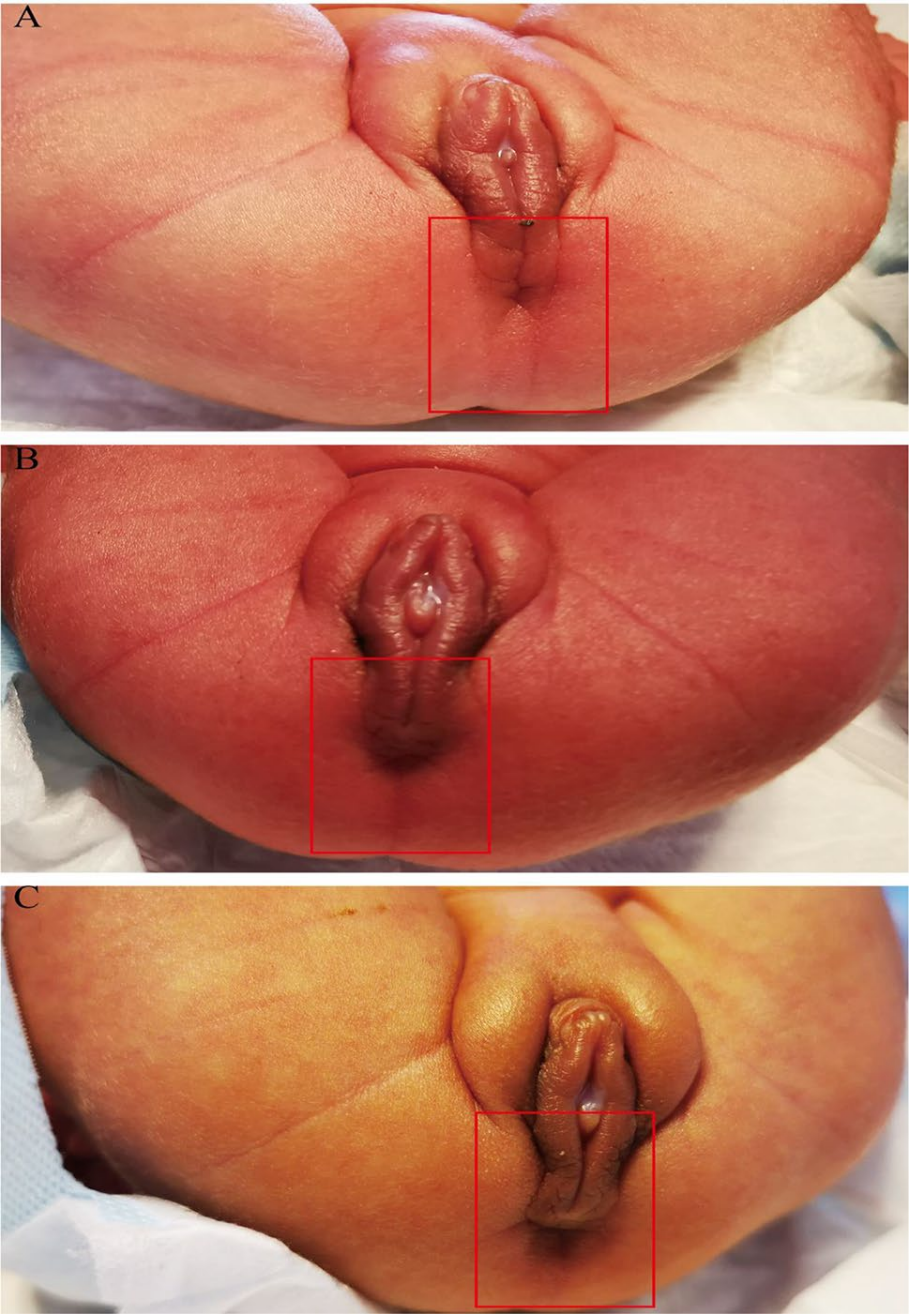
The triplets presented clinically as having anal agenesis. Supine photographs showing the clinical presentation of the triplets without anus. The eldest of the triplets (**A**), the second of the triplets (**B**), and the third of the triplets (**C**) in the supine position, red box indicated normal anatomical position without anal appearance

Due to the mother’s premature rupture of membranes, the triplets were delivered via an emergency cesarean section on the 4th day of the 34th week. During the intervention, it was confirmed that the triplets were dichorionic triamniotic. Following birth, all siblings were diagnosed with anal stenosis (Fig. 1) [10]. Additionally, the third triplet had syndactyly of the second and third left toes. Due to prematurity, they were immediately admitted to the neonatal intensive care unit (NICU). The triplets underwent chest X-ray, as well as abdominal and transfontanellar ultrasound, hearing screening by automated transient otoacoustic emission (aTEOAE), and automatic auditory brainstem response (aABR) which resulted normal. Cardiac ultrasound revealed pulmonary hypertension in the second and the third of the triplet. Abdominal radiography was also performed, revealing the presence of inflated and dilated intestinal loops. Thereafter, we noticed that all the triplets defecate through the vagina so we suspected rectovaginal fistula. Therefore, the posterior rectal advancement anoplasty was not performed at birth. However, limited by the conditions at that time, a barium enema was not performed, which is used to examine the location of the fistula in relation to the normal anal structure. By inquiring about their medical history, we were told that their father was born with imperforate anus, thumb malformations and hearing loss. He underwent anorectoplasty. He had moderate hearing loss and did not need hearing aids. Furthermore, during the family history investigation, another interesting finding emerged: the triplets’s grandmother was diagnosed with deafness and her brother had congenital anal atresia and finger deformities. Unfortunately, contact with him has been lost. Renal and cardiac US were not performed neither in the triplets’s father nor in the grandmother.

**Fig. 2:**
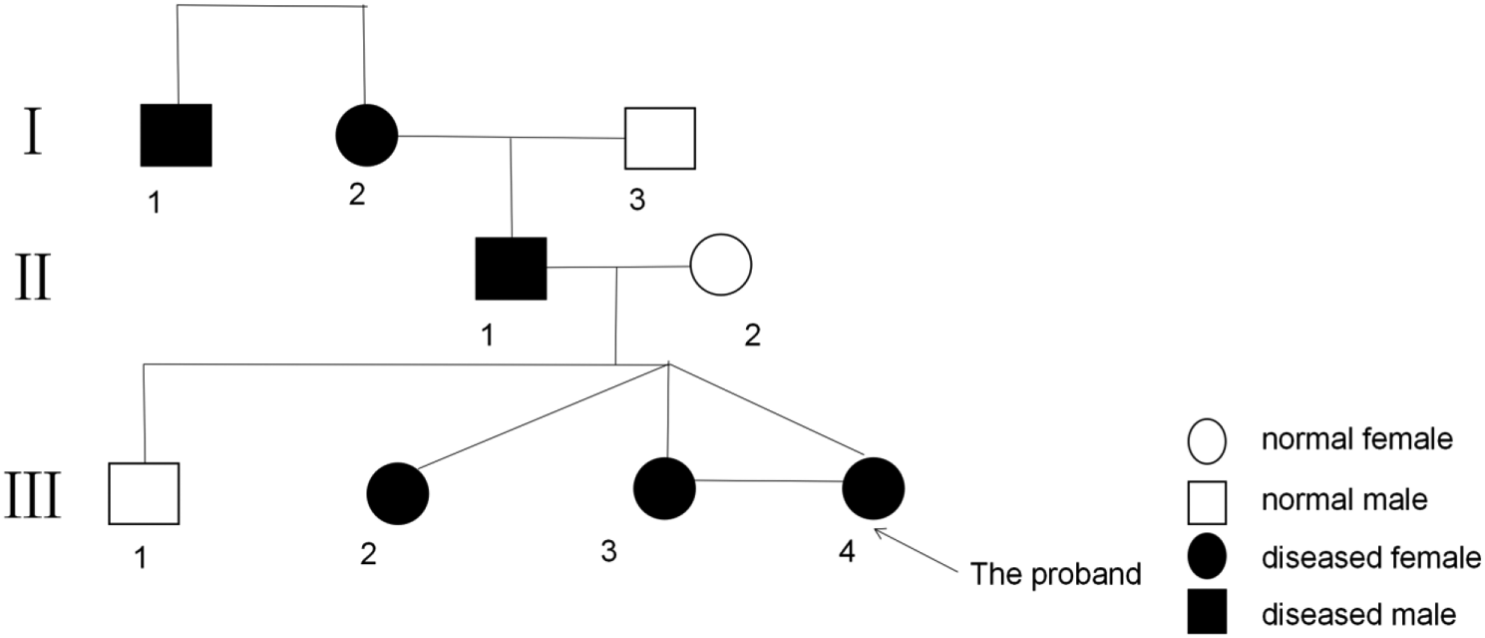
Pedigree of this family. The medical history was inquired about the disease status of family members.Square indicates male, circle indicates female, black indicates TBS patients and arrow indicates the proband (the third of the triplets)

The study describes 3 generations: grandmother affected by moderate hearing loss, father by moderate hearing loss, anal malformation, and thumb deformities, and triplets by anal atresia. The third one (III4) was chosen as the proband (Fig. 2). Peripheral blood samples were collected from all these individuals. Informed consent was obtained from the proband’s parents and all other family members. The study protocol was approved by the Shandong Provincial Hospital Affiliated to Shandong First Medical University Institutional Ethics Committee (SWYX: NO.2023 − 386).

## Results

### Sequencing data analysis

Although a-CGH is recommended first to detect genomic rearrangements [11–14], due to various factors we chose the gene sequencing method which is widely used in clinics at present. Genomic DNA was extracted using the QIAamp Blood Midi Kit from QIAGEN, located in Valencia, California. Paired-end reads of 150 bp were generated using an Illumina Novaseq6000 sequencer (Illumina, San Diego, CA, USA) and DNBSEQ (DNBSEQ-T7) (MGI, Shenzhen, China). The raw sequencing data was stored in FASTQ format. Quality control filters were applied to remove low-quality reads. After combining and splicing the clean reads using the second-generation sequencing analysis platform provided by MyGenostics, the coverage and sequencing quality of the target region were assessed. Subsequently, the flash analysis platform was utilized to examine the pathogenicity of the variants, and potential *loci* for these variants were identified. Furthermore, the pathogenicity of variation *loci* was evaluated according to the standards and recommendations set by ACMG (American College of Medical Genetics and Genomics). Sanger sequencing was employed to confirm potentially harmful mutations in all family members. An Applied Biosystems ABI 3730 analyzer was used for sequencing the mutations, and the results were compared with those obtained through capture sequencing.

**Fig. 3:**
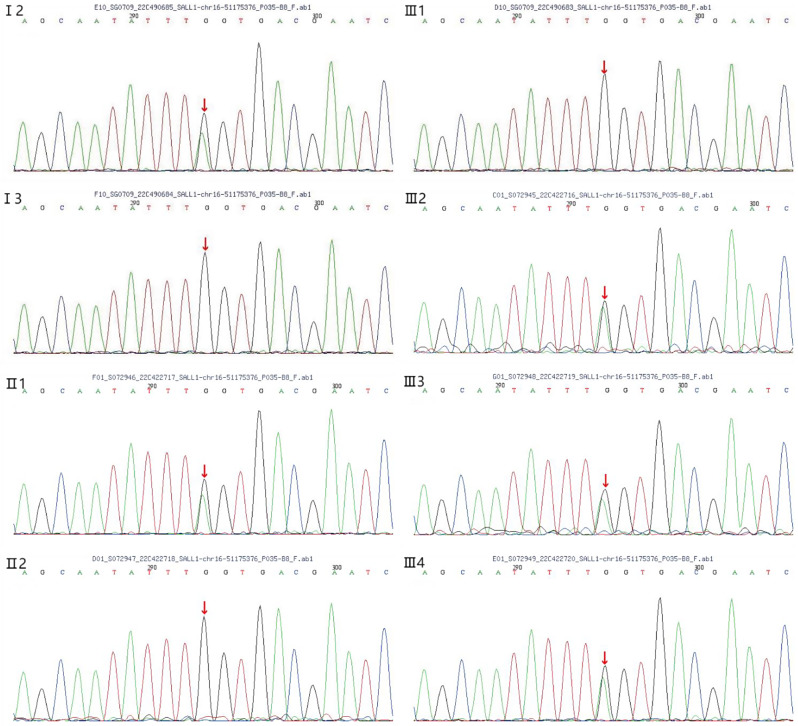
Sanger sequencing analysis of *SALL1* gene in genomic DNA from the family. The arrow indicates the mutation site (chr16:51175376: c.757 C > T p.Gln253* exon2, NM_002968). The proband (III4), her sisters (III2, III3), her father (II1) and her grandmother (I2) carried the mutation. Other family members did not have the mutation

**Fig. 4:**
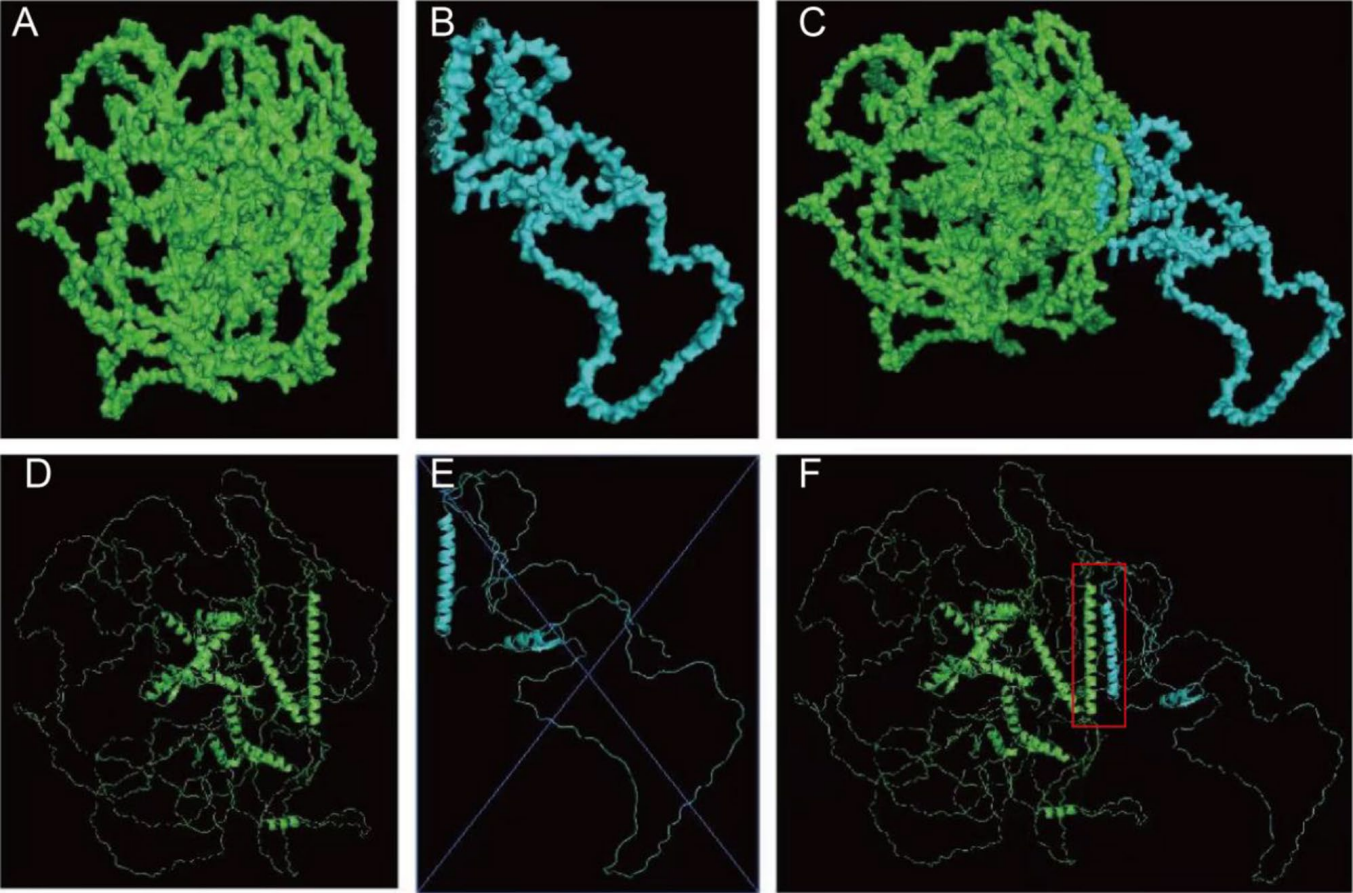
Predicted structures of human wildtype and mutant SALL1 protein (Gln253*). The AlphaFold structure predictions of human wildtype (green, **A**,**D**) and mutant (blue, **B**,**E**) SALL1 protein (Gln253*). The orthogonal views were visualized by PyMol program. The interaction of the wildtype and mutant SALL1 was highlighted in the red box (**C**,**F**)

Whole-exome sequencing analysis was performed on all of the family members. Molecular analysis revealed a heterozygous C to T transition at nucleotide position 757 in exon 2 of the *SALL1* (NM_002968) gene (Fig. 3). This transition was predicted to result in the substitution of the Gln253 codon with a premature stop codon (p.Gln253*), resulting in truncated SALL1 proteins. The structure of wildtype and mutant SALL1 protein (p.Gln253*) were predicted by AlphaFold (34,265,844). The structures were visualized by PyMol, and shown as orthogonal views (Fig. 4). It shows that the SALL1 (p.Gln253*) is a truncated protein that retains the glutamine-rich domain. Molecular docking, which has proven versatile for identifying protein-ligand interactions (23,508,189), was performed to predict the potential binding sites between wildtype and mutant SALL1 proteins. The results showed that the glutamine-rich domain forms a long alpha helix, through which the mutant protein could interact with wildtype SALL1 protein, with the lowest binding free energy. The binding site (red box in Fig. 4) was between the two glutamine-rich domains of wildtype and mutant SALL1 proteins, which is similar with previous reported [7, 15–20]. The interaction might exert steric hindrance effects to the wildtype SALL1 protein. This variant, although not listed in major mutation databases, cosegregated with the disease in six affected family members but was absent in unaffected ones. Following ACMG guidelines, c.757 C > T p.Gln253* is classified as pathogenic (PVS1 + PM2_supporting + PP1).

### Clinical prognosis

Follow-up was performed at a pediatric clinic one month after the surgery. The anal appearance of the triplets was satisfactory, and urination and defecation were normal without any signs of constipation [13]. At their corrected age of 1 year, they are thriving, exhibiting good weight gain, and experiencing no feeding or bowel difficulties. There were no reports of urinary tract infections during the outpatient follow-up period. Moreover, no surgical complications, such as anal stenosis, were observed. Clinical evaluation of their growth according to WHO growth charts and their development were followed. The triplets had regular evolution. Clinical laboratory indicators such as hemoglobin, alkaline phosphatase, and 25(OH)D were within normal ranges. Overall, their general growth and living conditions were satisfactory, similar to those of normally developing children [21]. 

## Discussion

As of March 2022, HGMD has documented 116 *SALL1* mutations, such as frameshifts, nonsenses, gross deletions, and splice variants [7]. Most of the reported mutations in TBS patients are frameshift or nonsense mutations that reside within a mutational hotspot region spanning from nucleotide 764 to 1565. These particular frameshift or nonsense mutations within the mutation hotspot region usually result in classical or more severe TBS phenotypes. Conversely, clinical manifestations due to haploinsufficiency of *SALL1* tend to be relatively mild. (22–23) Furthermore, genetic anticipation has been observed in certain families with Townes-Brocks syndrome, with an increase in clinical severity across generations [24]. The novel mutations (c.757 C > T p.Gln253* exon2) identified in this study were located in the hotspot mutation region of *SALL1*. The affected individuals in the first generation (the proband’s grandmother) exhibited deafness, while those in the second generation (the proband’s father) manifested polydactyly, deafness, and anal abnormalities. The third generation (comprising the proband and her sisters) displayed syndactyly of the second and third left toes, and anal abnormalities. This clinical presentation is consistent with findings from previous studies [24]. 

According to earlier studies, *SALL1* heterozygous deletions can cause Townes-Brocks syndrome or at least a milder form of the condition [8]. Indeed, the inheritance pattern of TBS follows autosomal dominant inheritance, meaning that a single copy of the mutated gene is sufficient to cause the disorder. In this case, the molecular basis of the disease lies in a specific gene called *SALL1*, which is located at 16q12.1 and encodes a zinc finger protein involved in transcription regulation [7, 23]. Mutations in the *SALL1* gene, typically heterozygous mutations, play a crucial role in the development of TBS. These mutations contribute to the syndrome through a combination of a dominant negative effect, where the mutated protein interferes with normal cellular processes, and haploinsufficiency, where there is an inadequate amount of functional protein produced due to the mutation [25]. Other studies have confirmed that these truncated proteins exert their influence by negatively affecting heart and limb development via a dominant negative effect [26]. In this study, proband’s father and sisters had finger deformity.

Diagnosis of Townes-Brocks syndrome (TBS) involves a combination of clinical evaluation, imaging studies, and genetic testing to identify mutations in the *SALL1* gene [7]. During the diagnostic process, it is crucial to exclude other rare syndromes, such as VACTERL/VATER syndrome, cat’s eye syndrome, and Goldenhar syndrome, that have TBS overlapping signs [27–29]. Treatment is generally supportive and may involve surgical intervention for certain malformations or the use of hearing aids for hearing loss [6]. In this case, the primary presentation of the triplets was anal agenesis, and timely surgical treatment was performed by experienced medical team. Following surgery for anorectal abnormalities, children may experience constipation and fecal incontinence [30]. Remarkably, the patients described in this study did not experience any negative side effects such as urinary tract infections, fecal incontinence, or constipation after the surgical treatment. Furthermore, our research observed patients with TBS syndrome for more than one year and we intend to continue following up with the patients until they reach maturity. Due to the numerous clinical manifestations of TBS syndrome, triplets need a multidisciplinary follow-up to test their growth, and their neurocognitive and behavioral evolution.

There have been reports of Anorectal malformations (ARMs) in 1 in 2,000 to 5 in 10,000 cases [31]. It is more common among Asians. (32–33) While there appears to be a low rate of inheritance in family members, some individuals exhibited autosomal dominant inheritance patterns [31]. Our subsequent genetic analysis further confirmed that this case represents a typical instance of autosomal dominant inheritance. Reconstructive surgery and ongoing symptom management (e.g., treatment for persistent constipation, incontinence, recurrent infections, and psychosocial support) constitute the major aspects of ARM clinical management [34]. In this case, after a period of conservative treatment, surgery was performed, and the postoperative effect was ideal, allowing the triplets to defecate independently without known sequelae. Multiple pregnancies have become more common in recent years due to the development of reproductive techniques. Spontaneous multiple pregnancies are rarely observed [35]. Their incidence is approximately 1:7000 [36]. It is worth noting that the clinical variability described in the affected subjects may be linked to different types of variants or to potential epigenetic factors (they may play a more relevant role in case of multiple pregnancies). (37–38) Yang et al. [24] reported a case of a 2-month-old child with Townes-Brocks syndrome (TBS). This patient presented clinical symptoms similar to those observed in our reported case. However, unlike us, the child’s parents were consanguineous and had a mutation in the *PTPRQ* gene, which has been associated with nonsyndromic hearing loss. Innoceta AM, et al. [39] described a case of a 7-year-old girl who not only exhibited symptoms similar to our current case but also presented with a ventrally positioned anus, pantonal sensorineural hearing loss (prevalent in the left ear) of mild-medium severity. Furthermore, she experienced developmental delay and speech delay. It is noteworthy that approximately one third of TBS cases reported in the literature involve some level of postnatal growth retardation [3, 40]. As a result, it is crucial for us to closely monitor the growth and development of these children.

We have successfully identified a novel mutation that has not been previously reported in individuals with TBS, thereby offering new genetic information crucial for genetic counseling and family screening. By reporting this novel variant, we have expanded the existing genetic variation spectrum of TBS and enriched the data available for understanding the molecular mechanisms of the disease. This particular locus has demonstrated an association with the clinical phenotype of TBS, which suggests that our findings could contribute to a better understanding of the disease’s presentation patterns, early diagnosis, and treatment guidance. However, it is important to note that our study focused on a single family, necessitating consideration of factors such as genetic heterogeneity and population differences that may influence the results. Additionally, our study did not take into account the interaction between genetic and environmental factors, including the potential influence of other genetic variants or environmental factors on the development of TBS. Further research is needed to explore these aspects.

## Conclusions

In this study a novel heterozygous mutation of *SALL1* (chr16:51175376: c.757 C > T p.Gln253* exon2, NM_002968) has been identified in a Chinese family with TBS typical phenotype. These findings enrich knowledge about TBS and contribute to better clinical management and genetic counseling. To gain more specific insights into the outcomes, long-term follow-up is required. Further studies are needed to understand the correlation between genotypes and phenotypes.

## Data Availability

All data generated or analyzed during this study are included in the article, further inquiries can be directed to the corresponding author.

## Abbreviations

SALLs: Spalt-like transcription factors
TBS: Townes-Brocks syndrome
PTCs: Premature termination codons
ARMs: Anorectal malformations
NT: Nuchal translucency
NICU: Neonatal intensive care unit
ACMG: American college of medical genetics and genomics
PRAA: Posterior rectal advancement anoplasty
PH: Pulmonary hypertension
TRV: Tricuspid regurgitation velocity
mPAP: Mean pulmonary artery pressure
